# Molecular Profiling of Endometrial Cancer: An Exploratory Study in Aotearoa, New Zealand

**DOI:** 10.3390/cancers13225641

**Published:** 2021-11-11

**Authors:** Claire E. Henry, Khoi Phan, Elena J. Orsman, Diane Kenwright, Michelle C. Thunders, Sara K. Filoche

**Affiliations:** 1 Department of Obstetrics, Gynaecology and Women’s Health, University of Otago, Wellington 6021, New Zealand; elena.orsman@otago.ac.nz (E.J.O.); sara.filoche@otago.ac.nz (S.K.F.); 2 Southern Community Laboratories, Wellington 6021, New Zealand; hkhoiphan@gmail.com; 3 Department of Pathology and Molecular Medicine, University of Otago, Wellington 6021, New Zealand; diane.kenwright@otago.ac.nz (D.K.); michelle.thunders@otago.ac.nz (M.C.T.)

**Keywords:** endometrial cancer, molecular, subtype

## Abstract

**Simple Summary:**

The incidence rate of endometrial cancer is rising globally. The molecular subtypes of endometrial cancer are independent of histology and have strong prognostic value in high-risk cancer. However, molecular profiling has not made it to clinical practice in Aotearoa, New Zealand. Therefore, we aimed to explore the feasibility of molecular profiling to examine the distribution of endometrial cancer subtypes and identify areas of need for implementation.

**Abstract:**

Background: Aotearoa, New Zealand, has one of the fastest-rising rates of endometrial cancer (EC) worldwide, increasing particularly in younger Māori and Pasifika women. There is a move towards using molecular profiling to direct treatment for each EC subtype. Aim: This study aimed to explore the molecular profiling of primary EC tissue in Aotearoa. Methods: We used the PORTEC guidelines for the molecular subtyping of 90 patients’ samples into four categories: *POLE*-mutated, p53 abnormal, mismatch repair deficient (MMRd) and no specific molecular profile (NSMP). The *CTNNB1* mutation and L1CAM expression were also included in the analysis. *POLE* and *CTNNB1* mutations were analysed using targeted next-generation sequencing (NGS). Novel mutations were assessed using VarSome. MMRd, L1CAM and p53 abnormalities were analysed using immunohistochemistry. Results: In total, 15 samples were MMRd, 9 were p53 abnormal, 8 were *POLE-*mutated and the rest (56) were NSMP. Eleven samples had exon 3 *CTNNB1* mutations and eleven novel *POLE* mutations were described. Conclusion: Surrogate markers for *POLE* mutations should be investigated. The validation of *POLE* variants and CTNNB1 mutations as part of an Aotearoa-based molecular panel is warranted.

## 1. Introduction

Incidence rates of endometrial cancer (EC) are rising globally; however, Aotearoa, New Zealand, has one of the fastest-rising rates of EC. The majority of Aotearoa’s population is of European descent (70.2%), with the indigenous Māori being the largest minority (16.5%), followed by people of Asian ethnicity (15.1%), and Pacific Islanders (8.1%) (per the 2018 census). There are persistent and stark inequities in access to healthcare and health outcomes, the impacts of which vary with ethnicity and socioeconomic status [1,2]. EC patients are subject to the effects of these inequities; women who identify as Māori or Pasifika experience the greatest burden from this disease and the rapid increase in diagnosis is observed primarily in younger age groups [3,4]. Whilst the scientific literature continues to characterise cancers by genomic classification, clinical translation of these advances is not a reality in Aotearoa. In 2013, the Cancer Genome Atlas (TCGA) project identified four molecular classifications of EC [5], which were then simplified into the Proactive Molecular Risk Classifier for EC (ProMisE) guidelines for ease of clinical testing [6]. These guidelines distinguished: mismatch repair deficient (MMRd), p53 abnormal (p53abn), *POLE-*mutated (POLE) and non-specified molecular profile (NSMP) subtypes. To determine most of these profiles, immunohistochemistry (IHC) can be used. However, *POLE* mutations still need to be confirmed via DNA sequencing [7].

There has been increased interest driving the use of molecular classification in clinical practice [8] since the PORTEC-3 trial results showed that molecular classification has a strong prognostic value in high-risk EC [9]. Women with p53abn tumours had significantly improved survival rates when treated with adjuvant chemo-radiation therapy, whilst women with *POLE-*mutated EC had incredibly good prognoses regardless of treatment arm (chemotherapy or chemo-radiation therapy). However, whilst MMRd IHC has been in routine clinical practice in Aotearoa since 2017 (mainly for Lynch syndrome), p53 IHC and *POLE* testing have not been routinely available.

The pathogenicity of *POLE* mutations has been confirmed in the most common variants of EC; however, challenges around interpreting novel variants arise when this test is applied to clinical use. Authors Leon-Castillo et al. [10] set out to provide guidance around classifying novel somatic mutations based on a tumour mutation burden (TMB) score, thereby facilitating the implementation of *POLE* testing. This may not be feasible for clinical use as only hotspot mutations will be investigated.

Before changes to patient treatment pathways can be made, we firstly need to understand the molecular profile of EC in Aotearoa and then, secondly, determine whether testing is feasible and relevant. There are no surrogate markers of *POLE* mutation status; though some researchers have attempted to histologically identify differences in immune components such as tumour infiltrating lymphocytes (TILs) to indicate POLE status (evidence has suggested that an increase in these populations can indeed indicate *POLE* status) [11,12]. Therefore, this paper aims to pilot the molecular classification of EC in New Zealand women as compared to PORTEC3 data.

## 2. Materials and Methods

### 2.1. Cohort

This retrospective study was approved by the Health and Disability Ethics Committee (HDEC 19CEN146). Formalin-fixed paraffin-embedded (FFPE) tissue blocks of 90 ECs diagnosed between 2015 and 2017 were retrieved from a New Zealand tertiary hospital. The inclusion criterion was adenocarcinoma of any histology, and equal samples were sourced from Māori, Pasifika, and NZ–European ethnicities. Curetting/pipelle biopsy samples were prioritised due to their better fixation for IHC and higher clinical relevance; however, if these were not available, a block of the resected primary tumour was chosen. Tumour stage and grade were taken from the diagnosis at the time of resection.

### 2.2. Molecular Profiling

IHC staining for p53 and MMR proteins (MLH1, MSH2, MSH6 and PMS2) was performed at Southern Community Laboratories (SCL). IHC was performed on the Ventana Benchmark Ultra; the detection system used was a 3-step polymer horseradish peroxidase (HRP) DAB kit (OptiView DAB) from Roche (760–700)—two tests (MSH2 and PMS2) also required tyramide signal amplification (860–099) steps. Heat-induced epitope retrieval was performed using CC1 (950–224), a high-pH Tris-based solution. All antibodies were incubated at 36 degrees Celsius and diluted in Roche antibody diluent ADB250 (F17676Z). All samples were then counter-stained with hematoxylin II (790–2208) and bluing reagent (760–2037) for 4 min each. Antibody clones, dilution and the antigen retrieval method are outlined in Table S1. Being an antibody for research purposes, L1CAM was validated using known positive tissue (such as kidney and colorectal adenocarcinoma, mesothelioma and ovarian serous carcinoma), and optimised for working conditions of high pH antigen retrieval for 56 min and primary antibody incubation for 32 min. In some cases, IHC was repeated on multiple blocks from the same patient as some samples had insufficient staining, possibly due to autolysis (Figure S1).

DNA extraction was performed using the Illumina AmpliSeq Direct FFPE DNA kit (Illumina, San Diego, CA, USA, #20023378); however, the QIAamp DNA FFPE Tissue Kit (Qiagen, Valencia, CA, USA, #56404) was used when samples were small/low quality. Whole sections (curettings) or tumour sections (primary resection) of up to three FFPE block sections (each 10 µm in size) were used. The final DNA concentration was assessed fluorometrically (Qubit DNA HS assay, Thermo Fisher, Waltham, MA, USA). According to manufacturer instructions, 10–100 ng of DNA is required for library preparation. For samples with high quantities, 100 ng was used for each primer pool. For lower-quantity samples, the maximum amount of DNA was used for each pool, which fell within the range of the required starting sample. The DNA libraries were prepared using the Illumina AmpliSeq Library PLUS custom panel (coverage: 94.8% *POLE* and 99% *CTNNB1*) and quality control (QC) of the amplified products was measured using the DNA 1000 BioAnalyzer Chip (Agilent Technologies, Waldbronn, Germany). Subsequent sequencing of the pooled libraries was performed on the iSeq 100 platform (Illumina, San Diego, CA, USA). Data analysis (checking alignment to the hg19 human reference genome and variant calling) was completed using the DNA Amplicon pipeline on Illumina BaseSpace software. The mean total of PF reads was 616,829, the mean percentage of on-target aligned reads was 93%, the mean target coverage percentage was 86% and the mean SNVs reported was 16. *POLE* mutations were considered pathogenic based on previous interpretations of exonuclease domains (ClinVar). However, for variants of uncertain significance, VarSome (Kopanos et al., 2018) was used to determine potential pathogenicity.

### 2.3. IHC Analysis

**Figure 1:**
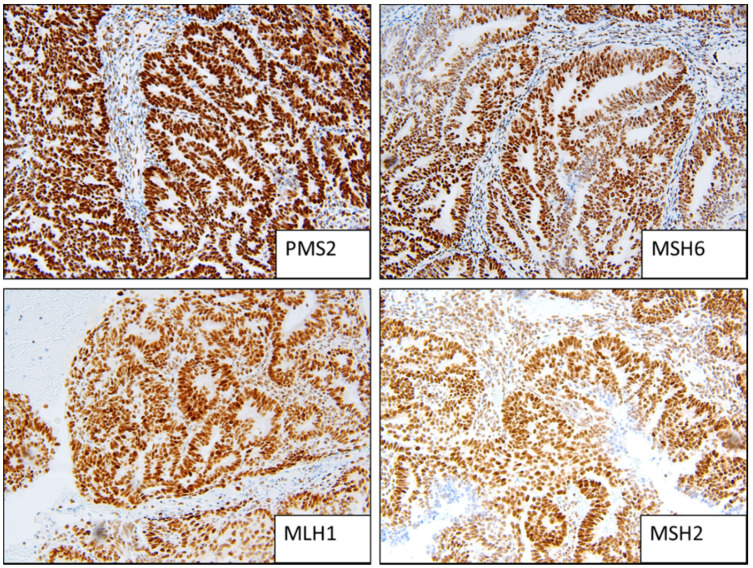
Example of MMR stable staining patterns (strong positive).

**Figure 2:**
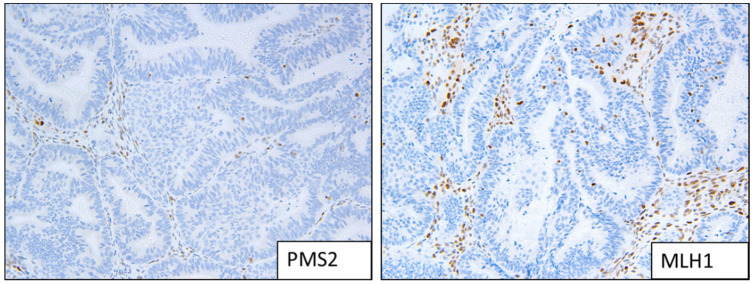
Example of MMRd staining patterns; loss of expression of MLH1 and PMS2.

**Figure 3:**
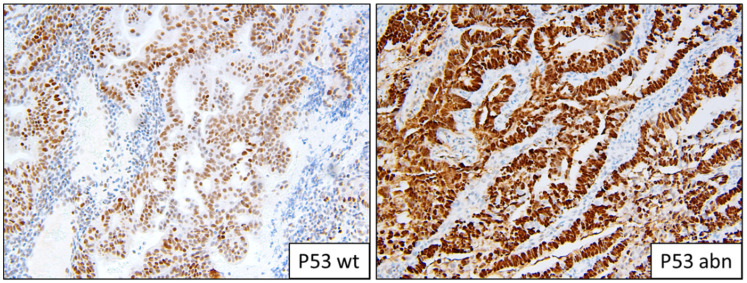
Example of p53 staining patterns; wild-type and abnormal overexpression.

Two people (a gynaecologic histopathologist and a research scientist) assessed the IHC independently (Figure 1, Figure 2 and Figure 3). Staining was deemed sufficient if both external (control tissue) and internal controls (non-neoplastic cells such as lymphocytes) were positive. Any discordance was discussed until an agreement was reached. p53 wild-type tumours were characterised by a mixture of negative, weak and strong cells, whereas p53abn was characterised by either diffuse strong staining or a complete absence of staining [13]. MMRd tumours were characterised by a complete absence of staining. L1CAM scoring was assessed on a 0 (absence of staining) to 3 (diffuse strong staining) scale (Figure 2). As per PORTEC 3 criteria, samples with a pathogenic *POLE* mutation superseded p53 abnormality/MMR loss and were therefore classified as *POLE*, and samples with a combination of loss of MMR and abnormal p53 were classified as MMRd.

### 2.4. Datamining

Based on the theory of an activated T cell infiltrate, the immune microenvironment of *POLE-*mutated EC was interrogated using the integrated repository portal for tumour–immune system interactions (TISIDB) [14]. We firstly screened for all *POLE* mutations, then curated the list to only uterine corpus endometrial carcinoma subsets (UCEC). This gave us outputs of differentially expressed lymphocytes, immunomodulatory molecules and chemokines in *POLE* Mut vs. wild-type samples. The most significant genes were then investigated in the context of overall survival using cBioPortal.

## 3. Results

Molecular testing was successful in 88 samples. IHC was not performed on two samples due to poor quality; multiple blocks were sampled (different representations of the primary tumour/cassettes); however, due to autolysis and the age of the blocks, IHC was not possible (Figure S1). The patient cohort characteristics are described in Table 1 (outcomes are recorded in Table S3). *POLE* and *CTNNB1* variants were classified as mutated if they were already reported as such in ClinVar. Variants that were likely pathogenic, or pathogenic as determined via VarSome, were documented. Benign or likely benign variants, determined by the type of mutation and location/pathogenicity algorithms, were excluded.

The 88 successful samples were then classified into one of four molecular subgroups (multiple-classifier ECs were identified and allocated a single molecular subgroup as previously described):

- MMRd: 15 (17%);
- P53abn: 9 (10%);
- *POLE*mut: 8 (9%);
- NSMP: 56 (64%).

Instances of multiple classifiers included three *POLE*mut samples that were also p53abn and two MMRd samples that also had pathogenic *POLE* mutations. We identified one sample that was stage IA, grade 1 and p53abn.

There were eight patients with high-grade, low-stage, high-risk endometrioid cancer (see Table 2). Of these, one was *POLE*mut, one was p53abn/*POLE*mut, four were p53abn and the other two were NSMP.

**Figure 4:**
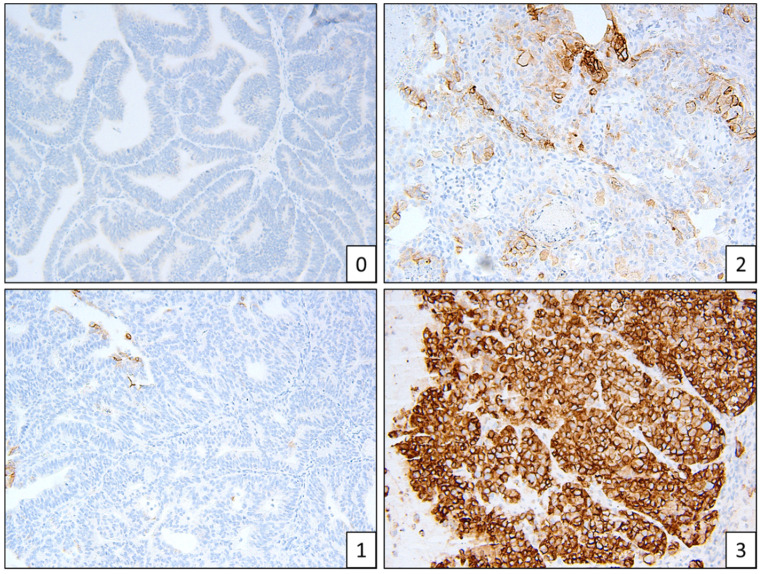
Example of L1CAM staining patterns, scores 0–3.

*CTNNB1* mutations seemed to be more frequent in Māori (*n* = 4) and Pasifika (*n* = 5) women, compared to NZ–European women (*n* = 2), although due to the small sample size this is not a significant difference (Table 3). All *CTNNB1* mutations were found in exon 3 (Table S2). Hotspot *POLE* mutations are listed in Table 4. L1CAM staining was low overall, with 79 of 88 stained samples (90%) having a score of 0–1 (Figure 4). L1CAM correlated with p53 mutations—if p53 was abnormal, L1CAM was more strongly expressed. L1CAM was noted to have focal and patchy staining.

### POLE Morphology

The utility of *POLE* testing will be realised when a surrogate marker can be used instead of sequencing. Already, specific exons are targeted for NGS (9–14) as these mutations are well described. However, from our data, we identified an additional 11 variants of uncertain but potential pathogenic significance (Table 5). Therefore, like others, we aimed to investigate whether increased TIL and nuclear pleomorphism could characterise these samples.

Only 3 out of the 10 samples deemed to belong to the *POLE* subtype had classic morphological markers; that is, increased TIL and nuclear pleomorphism. Two samples that were originally noted as low grade (grade 1) were subsequently identified as belonging to grade 3.

**Figure 5:**
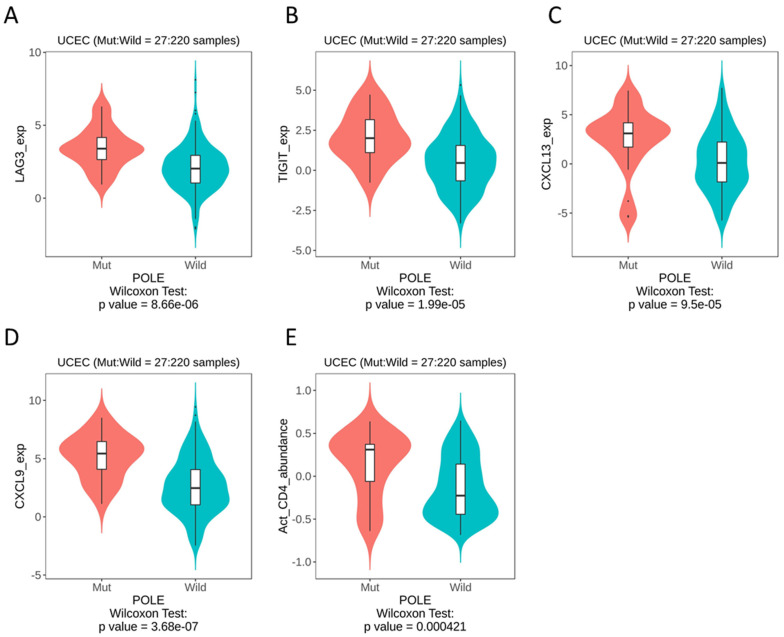
TISIDB datamining in UCEC, *POLE* mutation vs. wild-type, immune gene expression. (**A**) LAG3. (**B**) TIGIT. (**C**) CXCL13. (**D**) CXCL9. (**E**) Activated CD4 T cells.

Therefore, we looked to publicly available databases to investigate surrogate markers of *POLE*, based on the theory of an activated T cell infiltrate. Whilst the data set was derived from a small sample size (with a mut:wt ratio of 27:220), we were able to identify the most significant differences in LAG3 (*p* = 8.66 × 10^−6^), TIGIT (*p* = 1.99 × 10^−5^), CXCL13 (*p* = 9.5 × 10^−5^) and CXCL9 (*p* = 3.68 × 10^−7^) (Figure 5A–D). As expected, our datamining also showed significant differences in activated CD4 T cells (Figure 5E). When these immune genes were interrogated using cBioPortal, patients with alterations in TIGIT showed significantly improved overall survival rates compared to those with alterations in LAG3, CXCL13 and CXCL9 (Figure S2).

## 4. Discussion

This study was the first to explore molecular subtyping of EC in a New Zealand cohort. As expected for our cohort, most of the patients had a diagnosis of low-grade, low-stage endometrioid EC. We identified five samples with reportedly pathogenic *POLE* variants, and eleven potentially pathogenic variants; more research needs to be completed in order to validate these new variants. Interestingly, the only three classic-looking *POLE* samples (those showing increased TIL and nuclear pleomorphism) were those with variants of uncertain significance.

IHC was possible in 88 samples, with limitations caused by poor-quality staining on old and autolytic tissue. For the benefit of future molecular profiling, pipelle or curetting biopsies should be prioritised as these are not prone to poor fixation due to size, whereas large gynaecological samples are affected by this issue if they are not grossed in a timely manner. Failure to gross these large samples in a timely manner ultimately leads to tissue autolysis and poor staining and impacts on molecular typing. Furthermore, profiling on pre-surgical biopsies may influence the extent of surgical treatment, i.e., lymph node dissection, omental biopsy and/or washings for those with p53abn may be utilised, as appropriate. However, if DNA sequencing for *POLE* mutations is to remain in the profiling test, curettings may provide low-quantity material as there can be difficulty in getting sufficiently sized samples using a pipelle [15].

In this study, we included L1CAM and *CTNNB1* in the biomarker panel. There was no strong evidence to include L1CAM in the molecular profiling; most samples exhibited low L1CAM staining, and when it was strong, it correlated to those that were p53abn, which can be detected using p53 IHC instead. *CTNNB1* mutations were present in 11 samples, seemingly occurring more frequently in Māori or Pasifika women, although our sample size is too small to confirm this. *CTNNB1* mutations in exon 3 are associated with enrichment of the Wnt signalling pathway, and may characterise a subset of aggressive tumours associated with younger age and earlier stage [16]. In their 2020 study, Bigby et al. [4] reported that even though Māori and Pasifika women did not present with higher stage or grade, they experienced worse survival outcomes compared to NZ–European women. In our cohort, cancers in women with *CTNNB1* mutations did not have worse survival outcomes, but the average age of these women was less than that of the overall cohort (49 years compared to 59 years). Therefore, it may be worth validating *CTNNB1* to assess whether it may assist in risk stratification and improve equity in outcomes through an NZ-based molecular profile panel.

Further research into identifying a surrogate marker for *POLE* mutations is warranted. Currently, if molecular subtyping is to be completed in Aotearoa, some DHBs send samples overseas (for example, to Australia) to complete hotspot analysis (i.e., only addressing a number of exons). Sending samples overseas for profiling may further increase inequity in personalised care in numerous ways, including cost and data sovereignty [17]. Personalised medicine risks compounding, rather than reducing, inequities in cancer care and therefore the use of genomics must be carefully considered [18]. If *POLE* sequencing is to become mainstream, we need to have the capacity to have it accredited in our laboratories, the funds to undertake the testing and a robust method of delineating novel variants of uncertain pathogenicity. We attempted to look at other immune markers that may triage *POLE* samples using online databases. We found significantly upregulated molecules that may be used as surrogate IHC markers and warrant further investigation to determine relevance to NZ patients.

Lymphocyte activation protein 3 (LAG3) is a cell surface molecule expressed on activated T cells. As such, it fits within the theory of increased TILs correlating with *POLE* tumours. LAG3 has been investigated as an immune checkpoint protein [19] and numerous checkpoint inhibitors targeting LAG3 are currently in clinical trials. In a meta-analysis, the high expression of LAG3 was associated with improved survival in multiple solid tumours [20]. LAG3 expression can be measured using IHC, as demonstrated across multiple tumour types, with positivity described as >15 LAG3 positive TIL per 40x magnification hot spot field [21].

T cell immunoglobulin and ITIM domain (TIGIT) is another immune checkpoint molecule that has recently garnered attention as an emerging target in cancer immunotherapy [22]. TIGIT is a cell receptor that regulates T-cell-mediated tumour recognition, and as such fits the theorised *POLE-*mutated phenotype. One study in endometrial cancer linked resident tumour NK cell expression of TIGIT to disease severity [23]. Our datamining showed that TIGIT RNA expression is upregulated in *POLE*mut tumours which, based on the literature, would indicate worse outcomes [24,25]. It may be possible that this is over-ridden by *POLE* mutation and therefore women with tumours that have POLE and high TIGIT expression still have a favourable outcome (this effect is seen with p53). Furthermore, our survival analysis showed that patients with TIGIT alterations had the same exceptionally positive outcomes as those with *POLE* alterations, indicating that this molecule may warrant further investigation in our NZ panel or provide evidence for anti-TIGIT therapy for the treatment of late-stage *POLE*mut tumours.

Over 40 chemokines have been identified in humans. We found CXCL13 and CXCL9 were the most upregulated in *POLE-*mutated EC. It is well established that these chemokines play key roles in immune responses, including inflammation in cancer [26,27]. CXCL13-positive TIL are associated with a high mutation load and, in uterine cancer, B cells were predominantly observed in large aggregations in the tumour and stroma in 92% of POLE samples, compared to 48% of Microsatellite Stable tumours [28]. In ovarian cancer, high IHC expression (measured on a 0–3 scale) of the tumour cell CXCL9 was associated with doubled overall survival rates [29].

The main limitation of this study is the small sample size of 90. Further investigation, particularly when breaking cohorts down to different ethnicities and/or molecular subtypes, is important. However, this was the first pilot of molecular profiling in Aotearoa and these data will be useful in developing further investigations and guidelines for the uptake of this testing in clinical practice. To date, there is only one other study that has aimed to pilot the use of molecular stratification in endometrial cancer: Oberndorfer et al. [30] in Austria retrospectively published the first “real-world data”, from a small sample size of 40. The distribution of subtypes within that study was comparable to that found in ours. Twenty-three of these had shifted risk grouping due to molecular profile results; four patients were upstaged and nineteen were downstaged, which would have led to a change in treatment regime for twelve patients.

## 5. Conclusions

There needs to be more research focussed on the possibility of changing EC treatment based on a molecular subtype; larger-scale clinical trials, developed from the PORTEC-3 data, can inform such research. We identified that 11/90 women (12%) had *CTNNB1* mutations, suggesting that further investigation of this biomarker is needed in NZ women and that it may be appropriate to include it in an Aotearoa-specific genomic profiling panel. Overall, a large proportion of our cohort fell under the category of ‘non-specified molecular profile’. When breaking down subtypes and looking at treatment and outcomes, the major issues are around under-treating p53abn and early stage endometrioid tumours and over-treating those with *POLE* mutations. In our cohort, this accounted for around 8/90 patients (approx. 9%). Should it be, then, that *POLE* testing is only required for low-stage, high-grade tumours that harbour p53abn? In our cohort, this accounts for six women (7%). Based on NZ cancer statistics of newly registered uterine cases in 2018 (640), this would make a difference to an estimated 44 women each year. Further information in the form of prospective clinical trials is needed for these patients to safely receive de-escalated treatment based on their molecular profile.

## Figures and Tables

**Table 1:** Cohort characteristics.

		**Total *n* = 90 (100%)**	**Māori *n* = 30**	**Pasifika *n* = 30**	**NZ–Euro *n* = 30**
Age, years					
	Mean (range)		57 (29–78)	55 (33–72)	63 (37–85)
	<60		16	19	11
	>60		14	11	19
BMI					
	<30		3	0	4
	30–40		12	13	16
	>40		12	17	8
	Not recorded		3	0	2
Stage					
	IA		18	16	15
	IB		5	3	9
	II		0	2	3
	III		4	2	3
	IV		1	1	0
	Pathological stage not recorded		2	6	0
Histology					
	Endometrioid		27	29	28
	Serous		1	1	1
	Mixed		2	0	1
	Clear cell		0	0	0
Grade					
	Low (1–2)		25	27	25
	High (3)		5	3	5
Myometrial Invasion					
	<50%		20	19	15
	>50%		6	6	15
LVSI					
	Absent		23	22	19
	Present		3	3	11
Adjuvant Treatment					
	None		20	18	13
	Radiotherapy		6	10	13
	Chemotherapy		2	0	1
	Chemo-Radiation Therapy		1	0	3
Molecular subtype					
	MMRd		5	3	7
	p53ab		3	1	5
	*POLE*mut		3	3	2
	NSMP		18	23	15

**Table 2:** Molecular profile of high-risk EC (FIGO low stage, high grade).

**Histology**	**Stage**	**Grade**	**Subtype**	**Treatment**	**Outcome**
E/S	IA	3	p53abn/POLEmut	Chemo-Radiation Therapy	No recurrence
E	IA	3	NSMP	Pelvic radiotherapy	No recurrence
E	IB	3	p53abn	Pelvic radiotherapy	Recurrence 11 months
E	IA	3	p53abn	Brachytherapy	No recurrence
E/S	IA	3	p53abn	Alternative therapy	Recurrence 18 months
S	IA	3	p53abn	Brachytherapy	Recurrence 15 months
E	IA	3	POLEmut	Clinical followup only	No recurrence
E	IA	3	NSMP	Brachytherapy	No recurrence

E: endometrioid, S: serous, E/S: mixed.

**Table 3:** Pathogenic *POLE* and *CTNNB1* mutations.

***POLE*** **Mutations**	
Hotspot mutation	5
Uncertain significance	11
CTNNB1 mutations	
	11
L1CAM IHC score	
0	59
1	20
2	7
3	2

**Table 4:** Hotspot *POLE* mutations.

**HGVS Coding**	**HGVS Protein**	**Location**	**Sample Number**
c.1231G > C	v411L	exon 13	26
c.857C > G	P286R	exon 9	37
c.1099T > G	F367V	exon 11	74
c.1376C > T	S459F	exon 14	81
c.1376C > T	S459F	exon 14	84

**Table 5:** Novel *POLE* mutations likely to be pathogenic.

**HGVS Coding**	**HGVS Protein**	**Location**	**Sample Number**
c.5002G > A	G1668S	exon 38	25
c.3290C > T	A1097V	exon 27	26
c.3568G > A	E1190K	exon 29	29
c.5957delT	L1986Cfs13	exon 43	31
c.3501_3502insGGTCAAA	H1168Gfs11	exon 29	38
c.1337G > A	R446Q	exon 13	29
c.6160T > G	Y2054D	exon 45	37
c.430C > A	H144N	exon 6	37
c.154C > T	R52W	exon 2	37
c.1916G > A	R639H	exon 17	77
c.217G > A	D73N	exon 3	87

## Data Availability

Not available.

## Supplementary Materials

The following are available online at https://www.mdpi.com/article/10.3390/cancers13225641/s1, Figure S1: Example of insufficient staining patterns in autolysed endometrial tissue. Figure S2: Cbioportal output; Kaplan meier survival of altered immune genes identified in Figure 5; LAG3, TIGIT, CXCL13 and CXCL9. Table S1: IHC conditions. Table S2: CTNNB1 mutations. Table S3: Women who had recurrent endometrial cancer during the study period.
