# Supplementary material for: Molecular Profiling of Endometrial Cancer: An Exploratory Study in Aotearoa, New Zealand
Source: Cancers (Basel). 2021 Nov 11;13(22):5641. doi: 10.3390/cancers13225641 (PMC8615986; doi:10.3390/cancers13225641)
Supplement: Supplementary file 1 [file cancers-13-05641-s001.zip › cancers-1412114-supplementary.pdf]

Supplementary Materials: Molecular Profiling of Endometrial Cancer: an Exploratory Study in Aotearoa, New Zealand

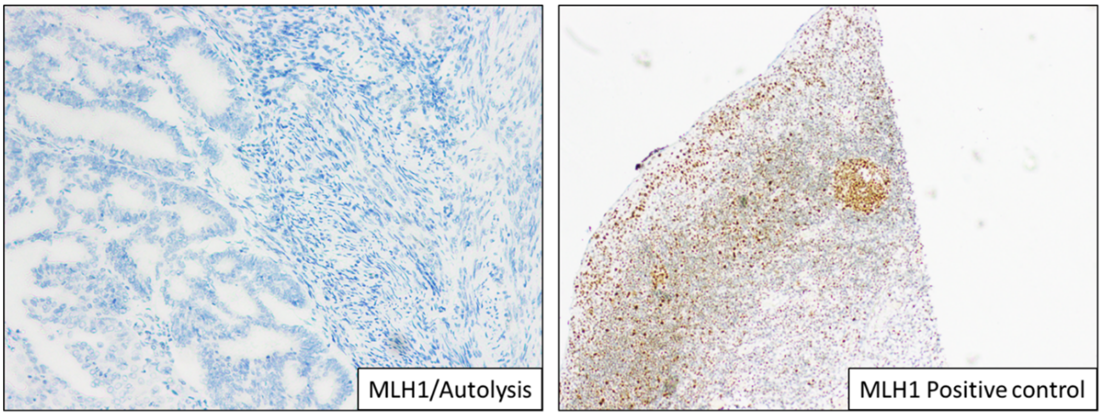

Figure S1. Example of insufficient staining patterns in autolysed endometrial tissue.

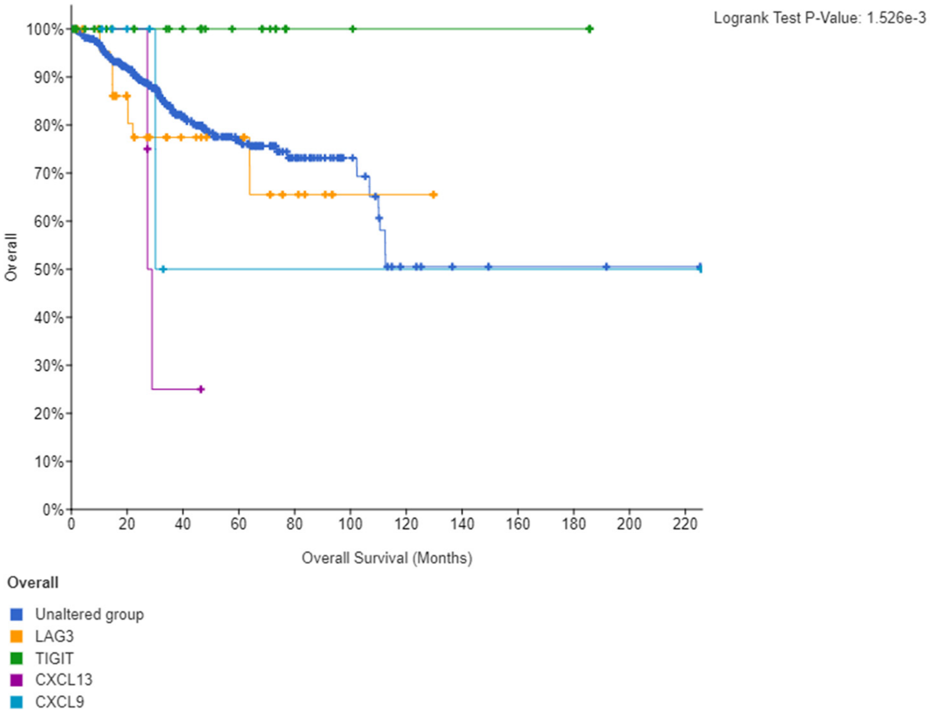

	Number of Cases, Total	Number of Events	Median Months Overall (95% CI)
Unaltered group	1241	189	NA
LAG3	47	11	NA
TIGIT	32	0	NA
CXCL13	4	3	27.27 (27.24 - NA)
CXCL9	8	2	30.11 (30.09 - NA)

Figure S2. Cbioportal output; Kaplan meier survival of altered immune genes identified in Figure 5; LAG3, TIGIT, CXCL13 and CXCL9.

Table S1. IHC conditions.

Antibody	MLH1	MSH2	MSH6	P53	PMS2	L1CAM
**Control**	Tonsil	Tonsil	Tonsil	Tonsil	Tonsil	Kidney
**RTU/TITRATION**	1:25	1:100	1:50	RTU	1:50	1:100
**Clone**	ES05	G219-1129	EP49	BP-53-11	EP51	14.1
**Reference**	M3640	286M-14	M3646	760-2542	M3647	L1
**Supplier**	DAKO (Denmark)	CELLMARQUE (USA)	DAKO (Denmark)	ROCHE (USA)	DAKO (Denmark)	Biolegend (USA)
**Detection**	Optiview DAB	Optiview DAB	Optiview DAB	Optiview DAB	Optiview DAB	Optiview DAB
**HIER**	CC1	CC1	CC1	CC1	CC1	CC1
**HIER Temp**	100	100	100	95	100	95
**HIER Time**	40	40	48	48	64	56
**AB Time**	32	16	32	20	24	32
**Amp**	N/A	Optiview AMP	N/A	N/A	Optiview AMP	n/a
**Amp Time**	N/A	4 minutes	N/A	N/A	4 minutes	n/a

Table S2. CTNNB1 mutations.

HGVS coding	HGVS Protein	Location	Sample number
c.110C>G	S37C	exon 3	22
c.101G>A	G34E	exon 3	26
c.110C>G	S37C	exon 3	32
c.101G>T	G34V	exon 3	39
c.110C>T	S37F	exon 3	35
c.94G>T	D32Y	exon 3	6
c.110C>T	S37F	exon 3	43
c.95A>T	D32V	exon 3	45
c.122C>T	T41I	exon 3	57
c.101G>T	G34V	exon 3	79
c.110C>T	S37F	exon 3	71

Table S3. Women who had recurrent endometrial cancer during the study period.

Histology	Stage	Grade	Subtype	Treatment	Recurrence (months)
E	IB	1	MMRd	Brachytherapy	33
E	IA	1	NSMP	clinical follow up only, radiotherapy upon recurrence	48
E	II	2	NSMP	Pelvic radiotherapy	8
E	II	2	MMRd	Chemoradiation therapy	27
E	IV	3	p53mut/POLE	Chemotherapy	passed away
E	IA	1	CTNNB1	clinical follow up only, radiotherapy upon recurrence	15
E	II	1	MMRd	Pelvic radiotherapy	19
E	IA	2	NSMP	clinical followup only	38
E	IA	2	NSMP	clinical follow up only	33
E	IVB	2	NSMP	declined treatment	passed away
E	IB	3	p53mut	Pelvic radiotherapy	13
E/S	IA	3	p53mut	declined treatment	18
S	IA	3	p53mut	Brachytherapy	15

© 2021 by the author. Licensee MDPI, Basel, Switzerland. This article is an open access article distributed under the terms and conditions of the Creative Commons Attribution (CC BY) license (https://creativecommons.org/licenses/by/4.0/).
